# Iatrogenic Right Ventricular Perforation Following an Endomyocardial Biopsy for Fulminant Myocarditis

**DOI:** 10.7759/cureus.99581

**Published:** 2025-12-18

**Authors:** Fumihiro Kitashima, Atsushi Harada, Yuki Hayashi, Keita Kamata, Masashi Tanaka

**Affiliations:** 1 Department of Cardiovascular Surgery, Nihon University School of Medicine, Tokyo, JPN

**Keywords:** cardiac tamponade, ecmo, endomyocardial biopsy, fulminant myocarditis, right ventricular perforation, surgical repair

## Abstract

Right ventricular (RV) perforation is a rare but potentially life-threatening complication of endomyocardial biopsy (EMB), particularly in patients with fulminant myocarditis, where myocardial edema increases myocardial fragility. We describe the case of a 50-year-old woman who developed acute RV free-wall perforation immediately after EMB performed for suspected fulminant myocarditis. Emergent pericardial drainage temporarily stabilized her hemodynamics, but ongoing hemorrhage necessitated urgent conversion to open surgical repair. A 3-mm perforation on the anterior RV wall was successfully sealed using a fibrin patch (TachoSil®) and a collagen-based hemostatic agent (Bolheal®), followed by extracorporeal membrane oxygenation support for circulatory stabilization. The patient recovered completely without recurrence and remained stable at the 10-month follow-up. This case highlights the need for early recognition of biopsy-related perforation, prompt surgical preparedness, and meticulous septal targeting during EMB, particularly in patients with myocarditis whose edematous myocardium is highly susceptible to mechanical injury.

## Introduction

Endomyocardial biopsy (EMB) remains the gold standard for the histopathological diagnosis of myocarditis and cardiomyopathies. Although generally safe when performed by experienced operators, major complications such as cardiac tamponade, ventricular perforation, and death occur in fewer than 1% of cases [1,2]. However, the reported incidence of right ventricular (RV) perforation varies widely across studies, ranging from 0.05% to 5%, depending on procedural techniques and biopsy instruments used [1,2].

Comparative studies have further clarified the relative safety of left ventricular (LV) and RV biopsy. Yilmaz et al. [3] reported that major complication rates were similar between LV and RV EMB (0.64% vs. 0.82%), while LV biopsy provided a higher diagnostic yield for myocarditis. Neves et al. [4] demonstrated that pericardial effusion and cardiac tamponade occurred more frequently after RV biopsy, likely due to the thinner RV free wall. Holzmann et al. [5] reported exceptionally low rates of major complications, 0.12% in the retrospective cohort and 0% in the prospective cohort, when EMB was performed by skilled operators.

We present a case of iatrogenic RV perforation following EMB performed for fulminant myocarditis, highlighting procedural risk factors, the importance of real-time imaging guidance, and multidisciplinary surgical management that resulted in a successful clinical outcome.

## Case presentation

A 50-year-old woman with a history of postoperative breast cancer who was undergoing hormonal therapy presented with dyspnea and bilateral leg edema. The patient experienced cardiogenic shock upon arrival and required inotropic support and intra-aortic balloon pumping. Laboratory data revealed elevated cardiac enzyme levels (Table 1).

**Table 1:** Laboratory findings on admission. Laboratory findings demonstrating markedly elevated cardiac enzyme levels.

Laboratory test	Result	Reference values
Creatine kinase (U/L)	4430	30–170
Creatine kinase-MB (ng/mL)	154	<5
Cardiac troponin I (ng/mL)	33.95	<0.04

**Figure 1:**
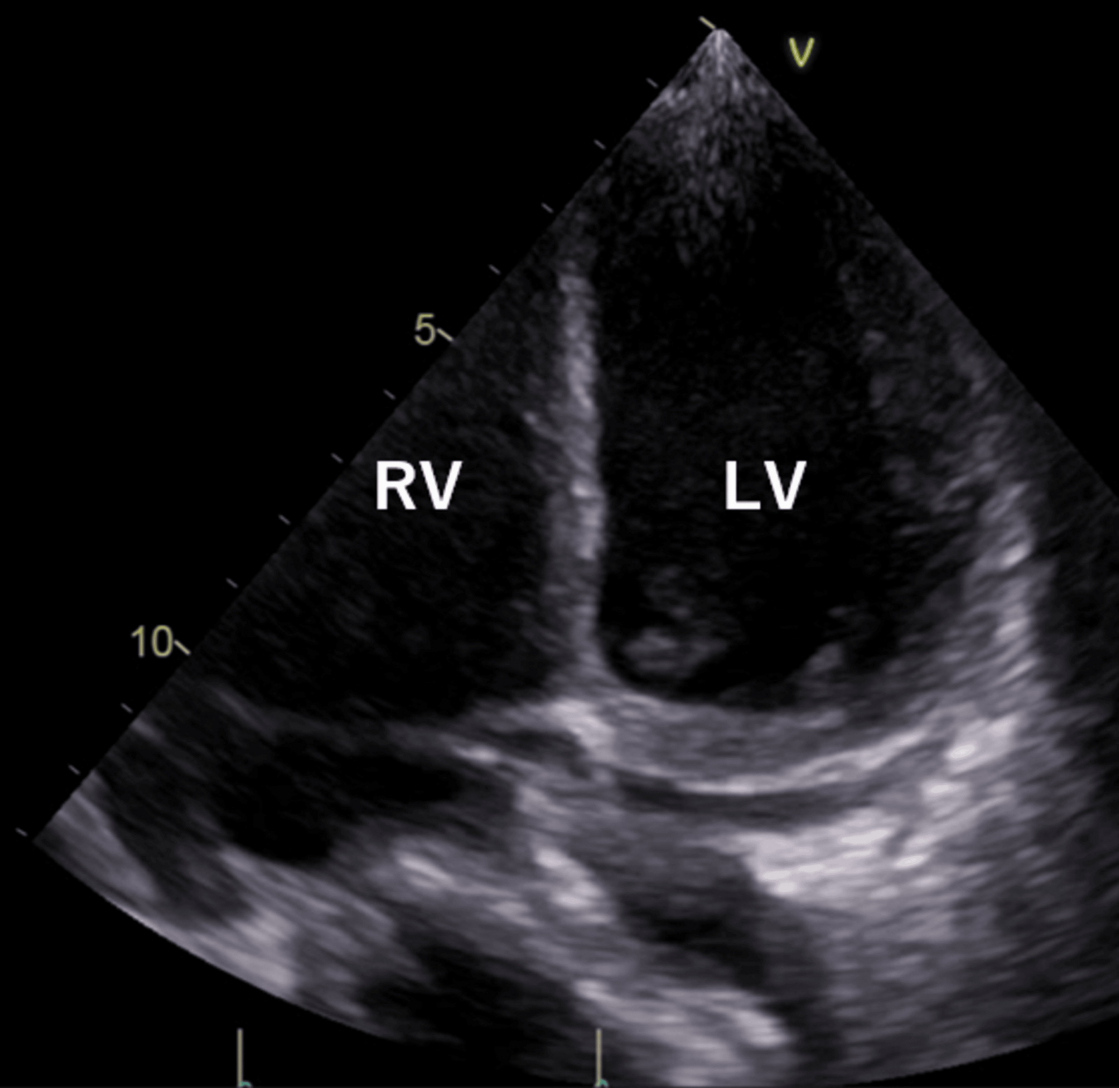
Transthoracic echocardiography. Transthoracic echocardiography on admission demonstrating diffuse left ventricular hypokinesis with a severely reduced ejection fraction (10–20%).

Echocardiography revealed diffuse hypokinesis (LV ejection fraction (LVEF) of 10-20%) (Figure 1).

**Figure 2:**
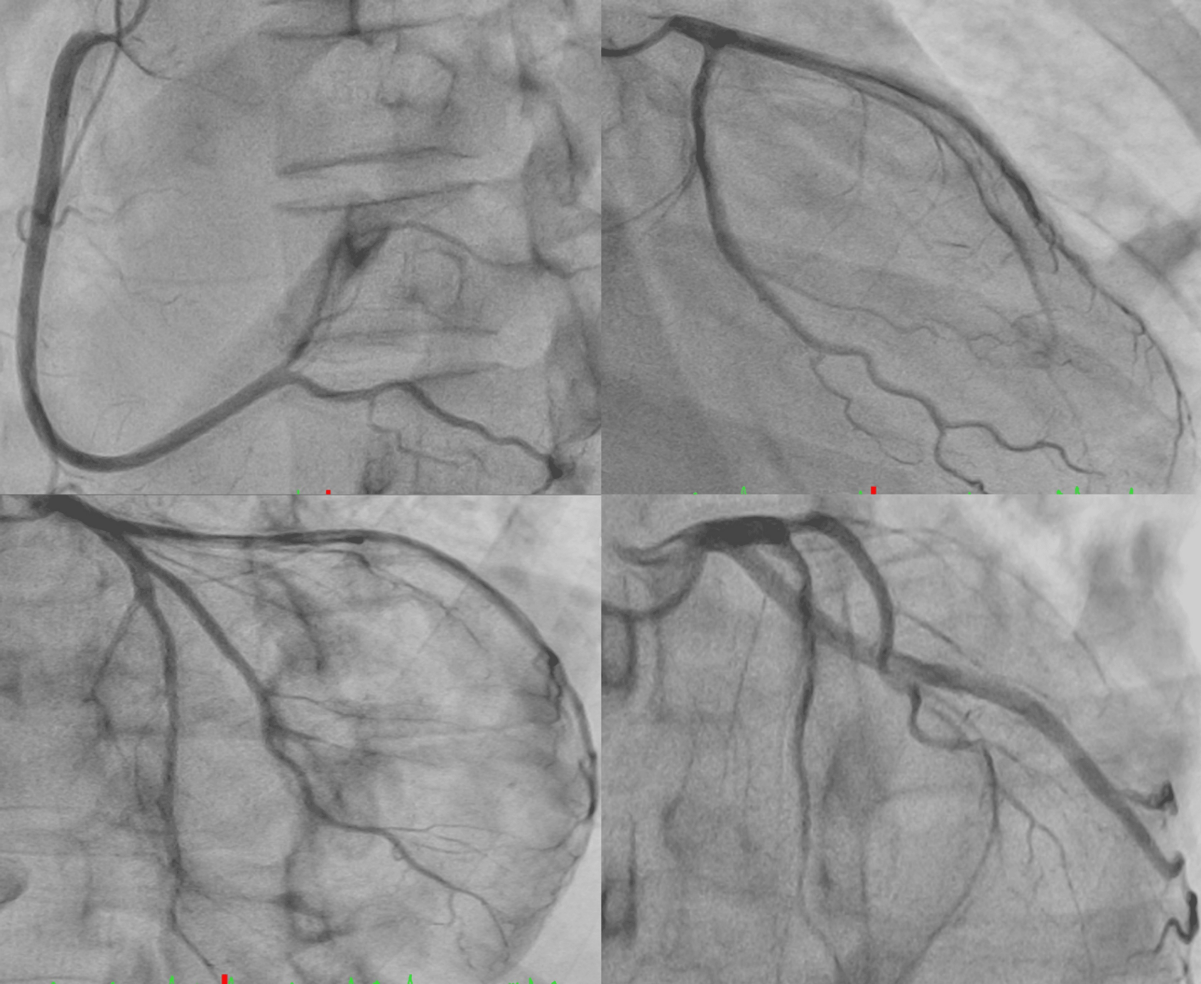
Coronary angiography. Coronary angiography on admission showing no significant stenosis in the major coronary arteries.

**Figure 3:**
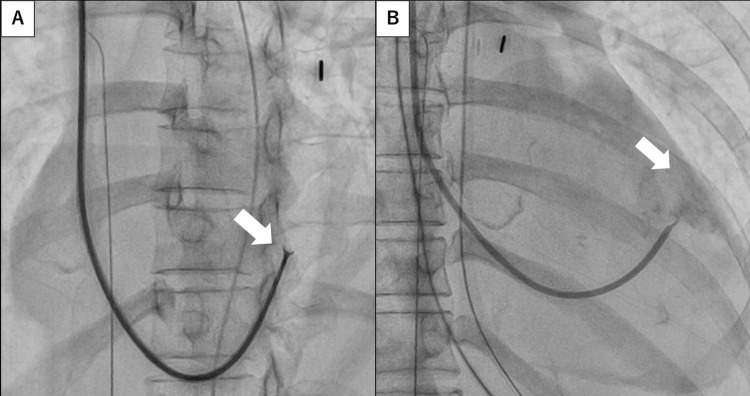
Fluoroscopic image and right ventricular angiography. (A) Fluoroscopic image showing endomyocardial biopsy performed via the right internal jugular vein, targeting the right ventricular interventricular septum (white arrow). (B) Right ventricular angiography immediately after biopsy demonstrating contrast extravasation into the pericardial space, consistent with free-wall perforation (white arrow).

Coronary angiography revealed no significant stenosis in the major coronary arteries (Figure 2), and fulminant myocarditis was suspected. On day four, EMB was performed via the right internal jugular vein under fluoroscopic guidance, targeting the interventricular septum (Figure 3A). The patient became hypotensive immediately after specimen retrieval. RV angiography revealed contrast extravasation into the pericardial space, confirming RV free-wall perforation (Figure 3B).

**Figure 4:**
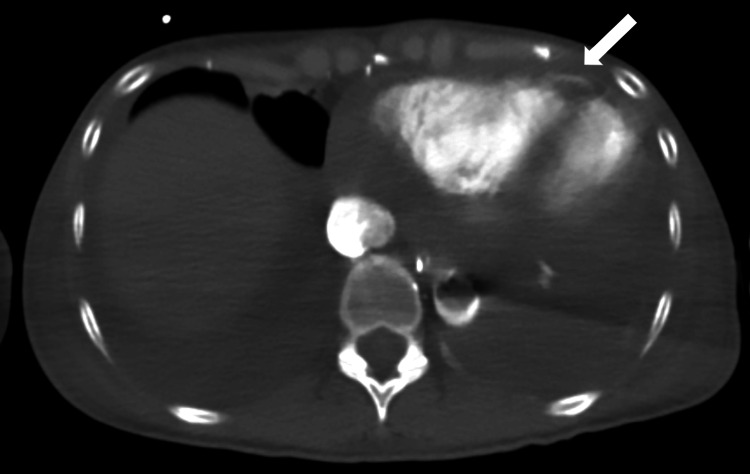
CT angiography. CT angiography showing active contrast leakage from the right ventricle into the pericardial cavity (white arrow), confirming persistent perforation with pericardial hematoma.

Emergent pericardial drainage yielded hemorrhagic fluid; however, bleeding persisted, and the blood pressure remained unstable. CT angiography revealed ongoing contrast leakage from the RV into the pericardial cavity (Figure 4).

**Figure 5:**
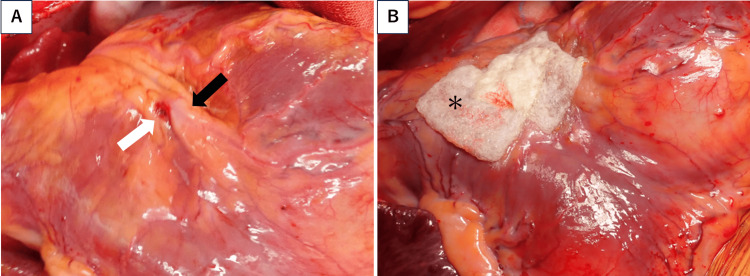
Intraoperative findings. (A) Intraoperative findings after hematoma evacuation, revealing a 3-mm perforation on the anterior right ventricular wall (white arrow) near the left anterior descending artery (black arrow). (B) Hemostasis achieved using Bolheal® and TachoSil® patches applied over the perforation site for reinforcement (asterisk).

Emergency median sternotomy was performed. Cardiopulmonary bypass was performed via femoral cannulation. The pericardium had a large hematoma. After hematoma evacuation, a 3-mm perforation was identified in the anterior RV wall near the left anterior descending (LAD) artery (Figure 5A). No apparent active bleeding was observed. Direct suturing risked coronary injury; thus, Bolheal® Tissue Sealant (KM Biologics, Kumamoto, Japan) and TachoSil® Tissue Sealing Sheet (CSL Behring, Tokyo, Japan) were applied for hemostasis and reinforcement (Figure 5B).

Venoarterial extracorporeal membrane oxygenation (ECMO) was instituted because of severely reduced LVEF (~10%). High-dose steroid pulse therapy was initiated postoperatively. The patient was successfully weaned from ECMO on postoperative day four (LVEF ≈ 20%) and discharged on day 50, with an LVEF of approximately 40%. At the 10-month follow-up, the patient remained stable without recurrence or complications.

## Discussion

RV perforation during EMB is an uncommon but critical complication, with a reported incidence ranging widely from 0.05% to 5% across published studies [1,6]. The mechanism typically involves mechanical trauma from the bioptome or inadvertent sampling of the RV free wall rather than the interventricular septum. In our case, perforation occurred during biopsy for fulminant myocarditis, in which myocardial edema and necrosis rendered the ventricular wall fragile and more susceptible to injury. These pathological conditions, combined with altered chamber geometry, likely contributed to the event despite careful manipulation.

The safety and diagnostic yield of EMB have been extensively investigated. Yilmaz et al. [3] reported in a large prospective study that major complication rates were comparable between LV and RV EMB (0.64% vs. 0.82%), yet LV sampling yielded a higher diagnostic accuracy for myocarditis. Similarly, Neves et al. [4] demonstrated through a meta-analysis that pericardial effusion and tamponade were more frequent after RV biopsy, reflecting the thinner RV wall and greater mechanical vulnerability. These findings highlight that although LV or biventricular biopsy may enhance diagnostic accuracy, operator experience and proper imaging guidance remain key determinants of procedural safety.

Holzmann et al. [5] analyzed large datasets and reported extremely low major complication rates, 0.12% in the retrospective cohort and 0% in the prospective cohort, when EMB was performed by skilled operators under fluoroscopic guidance. Drury et al. [6] emphasized that real-time echocardiographic or intracardiac echocardiography (ICE) guidance markedly reduces the risk of perforation by confirming septal targeting and enabling early detection of pericardial effusion. As a result, echo-assisted or hybrid fluoroscopic-ICE techniques have become preferred approaches for high-risk myocarditis cases.

In the present case, pericardial drainage achieved temporary hemostasis; however, initiation of venoarterial ECMO increased the risk of rebleeding and tamponade due to the need for anticoagulation. Therefore, open surgical hemostasis using topical agents (Bolheal® and TachoSil®) was performed for definitive control. Even small residual leaks can become fatal under ECMO support, and early surgical repair remains the most reliable approach when bleeding persists or mechanical circulatory support is required.

Most reported cases of RV perforation have occurred in post-transplant patients [7], in whom pericardial adhesions often limit hemorrhage. In contrast, non-transplant settings such as fulminant myocarditis lack adhesions, allowing rapid blood accumulation within the pericardial space and sudden hemodynamic collapse. This underscores the importance of prompt recognition, surgical readiness, and close coordination among cardiologists, cardiac surgeons, and intensivists to ensure a successful outcome.

From an educational standpoint, this case reinforces key preventive strategies: the necessity of multimodal imaging, gentle and controlled bioptome manipulation, precise septal targeting, and team-based procedural planning. Reporting such rare but instructive complications contributes to improved institutional protocols and enhances the overall safety of EMB in patients with vulnerable myocardium.

## Conclusions

RV perforation during EMB is rare but potentially life-threatening, especially in patients with fulminant myocarditis, where myocardial edema increases tissue fragility. This case highlights the importance of careful septal targeting, multimodal imaging guidance, and prompt recognition of hemodynamic deterioration. When perforation occurs, rapid surgical intervention and appropriate use of mechanical circulatory support, including ECMO, can be lifesaving. Early multidisciplinary collaboration is essential to ensure favorable outcomes in these high-risk scenarios.
